# Inhibition of T Cell Receptor Activation by Semi-Synthetic Sesquiterpene Lactone Derivatives and Molecular Modeling of Their Interaction with Glutathione and Tyrosine Kinase ZAP-70

**DOI:** 10.3390/molecules24020350

**Published:** 2019-01-19

**Authors:** Andrei I. Khlebnikov, Igor A. Schepetkin, Anarkul S. Kishkentaeva, Zhanar R. Shaimerdenova, Gayane A. Atazhanova, Sergazy M. Adekenov, Liliya N. Kirpotina, Mark T. Quinn

**Affiliations:** 1 Kizhner Research Center, Tomsk Polytechnic University, Tomsk 634050, Russia; aikhl@chem.org.ru; 2 Scientific Research Institute of Biological Medicine, Altai State University, Barnaul 656049, Russia; 3 Department of Microbiology and Immunology, Montana State University, Bozeman, MT 59717, USA; schepetkin@yahoo.com (I.A.S.); liliya@montana.edu (L.N.K.); 4 International Research and Production Holding “Phytochemistry”, Karaganda 100009, Kazakhstan; anar_kish@mail.ru (A.S.K.); arsenzhan@bk.ru (Z.R.S.); g-atazhanova@mail.ru (G.A.A.); arglabin@phyto.kz (S.M.A.)

**Keywords:** sesquiterpene lactones, ZAP-70, T cell receptor, extracellular signal-regulated kinase, calcium flux, molecular modeling, glutathione

## Abstract

A variety of natural compounds have been shown to modulate T cell receptor (TCR) activation, including natural sesquiterpene lactones (SLs). In the present studies, we evaluated the biological activity of 11 novel semi-synthetic SLs to determine their ability to modulate TCR activation. Of these compounds, α-epoxyarglabin, cytisinyl epoxyarglabin, 1β,10α-epoxyargolide, and chloroacetate grosheimin inhibited anti-CD3-induced Ca^2+^ mobilization and extracellular signal-regulated kinase 1/2 (ERK1/2) phosphorylation in Jurkat T cells. We also found that the active SLs depleted intracellular glutathione (GSH) in Jurkat T cells, supporting their reactivity towards thiol groups. Because the zeta-chain associated tyrosine kinase 70 kDa (ZAP-70) is essential for TCR signaling and contains a tandem SH2 region that is highly enriched with multiple cysteines, we performed molecular docking of natural SLs and their semi-synthetic derivatives into the ZAP-70 binding site. The docking showed that the distance between the carbon atom of the exocyclic methylene group and the sulfur atom in Cys39 of the ZAP-70 tandem SH2 module was 3.04–5.3 Å for active compounds. Furthermore, the natural SLs and their derivatives could be differentiated by their ability to react with the Cys39 SH-group. We suggest that natural and/or semi-synthetic SLs with an α-methylene-γ-lactone moiety can specifically target GSH and the kinase site of ZAP-70 and inhibit the initial phases of TCR activation.

## 1. Introduction

T cells play an essential role in inflammatory and adaptive immune responses, and the deregulation of T cell function can contribute to autoimmune disease [e.g., see [1]. The identification of specific inhibitors of T cell receptor (TCR) signaling could represent a potential for therapeutic treatments of autoimmune disease and has been of significant interest [2]. For example, several natural immunomodulatory compounds have been shown to alter TCR activation [3,4,5]. Recently, we found that the natural sesquiterpene lactones (SLs) arglabin, grosheimin, argracin, parthenolide, and estafiatin inhibited TCR activation [6]. SLs are natural products that exhibit a broad spectrum of biological activities, including antibacterial, antifungal, anticancer, anti-inflammatory, and immunomodulatory activities [7,8,9]. Indeed, arglabin has been used clinically for cancer treatment [10]. However, many natural SLs have poor biopharmaceutical properties and low bioavailability.

Structural modification has been pursued in order to increase SL potency and selectivity, improve physico-chemical, biochemical, and pharmacokinetic properties, and eliminate or reduce adverse effects [11]. To date, a number of SL derivatives with greater potency than their natural analogs have been synthesized [9]. For example, artesunate, a semi-synthetic derivative of artemisinin, has been reported to reduce the severity of experimental autoimmune encephalomyelitis by inhibiting the migration of pathogenic T cells to the CNS [12]. Likewise, several semi-synthetic parthenolide derivatives have been developed for their anti-leukemic activity [13,14]. In addition, synthetic modifications of SLs, including oxidation and esterification of the hydroxyl group, amination, reduction, and coupling of the α-methylene-γ-lactone moiety have resulted in variations in the anticancer activity of these compounds [13,14,15,16,17,18,19,20].

Here, we evaluated the capacity of 11 semi-synthetic compounds, derived from the natural SLs arglabin, argolide, 3β-hydroxyarhalin, and grosheimin, to alter the initial phases of TCR activation and showed that four of these compounds (α-epoxyarglabin, cytisinyl epoxyarglabin, 1β,10α-epoxyargolide, and chloroacetate grosheimin) inhibited TCR activation-induced Ca^2+^ mobilization and extracellular signal-regulated kinase 1/2 (ERK1/2) phosphorylation. These compounds also depleted intracellular glutathione (GSH). Because of the importance of the zeta-chain associated tyrosine kinase 70 kDa (ZAP-70) in TCR activation [21,22], we performed molecular docking studies using the three-dimensional structures of these natural compounds and their derivatives to explore possible binding modes with the ZAP-70 tandem SH2 module.

## 2. Results and Discussion

### 2.1. Isolation of Parent Natural SLs and Synthesis of Their Derivatives

**Figure 1:**
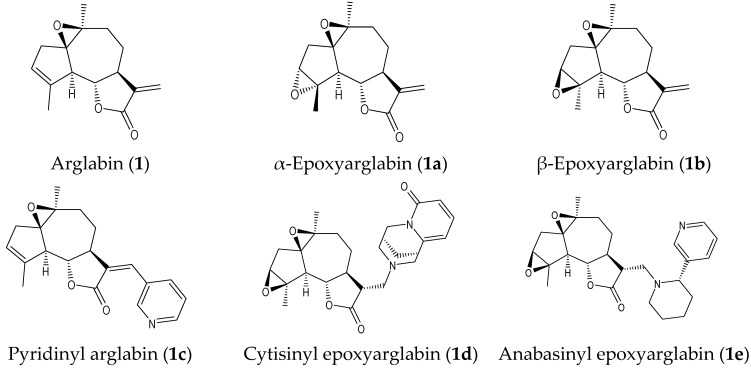

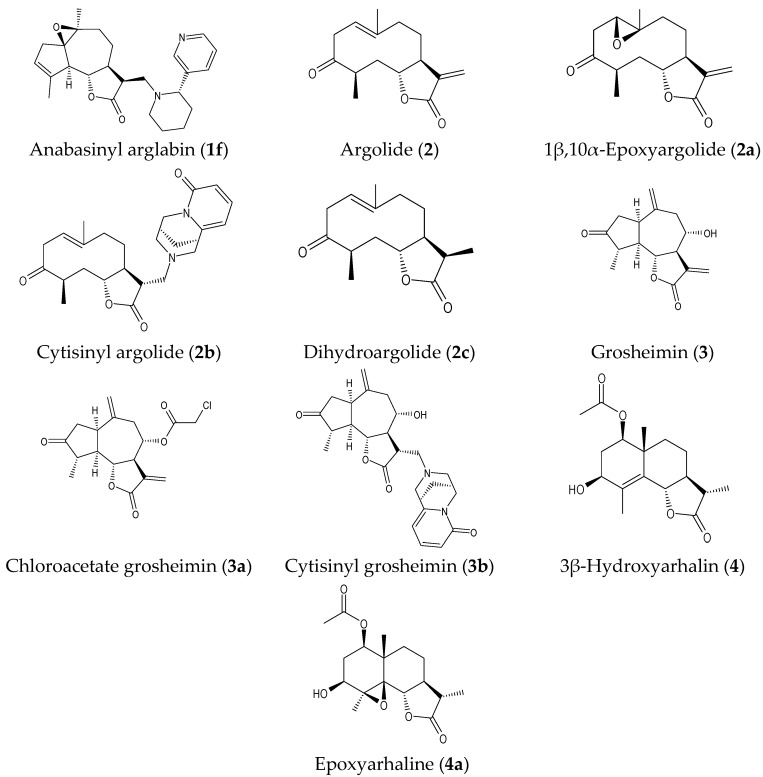
Chemical structures of natural sesquiterpene lactones (SLs) arglabin (**1**), argolide (**2**), dihydroargolide (**2c**), grosheimin (**3**), 3β-hydroxyarhalin (**4**), and their semi-synthetic derivatives **1a**–**f**, **2a**, **b**, **3a**, **b**, **4a**.

Arglabin (**1**), argolide (**2**), grosheimin (**3**), 3β-hydroxyarhalin (**4**), and dihydroargolide (**2c**) were isolated from different plants of the *Asteraceae* family (structures shown in Figure 1). Arglabin and argolide were isolated from *Artemisia filatovae* A. Kuprijanov sp. Nova [23], 3β-hydroxyarhalin was isolated from *Artemisia halophila* Krasch. [24], and grosheimin was isolated from *Chartolepis intermedia* Boiss [25]. Dihydroargolide (ketopelenolide B) was isolated from *Artemisia glabella* Kar. et Kir. [26].

Analogs of the isolated SLs, with the exception of chloroacetate grosheimin and pyridinyl arglabin, were synthesized using optimized reaction conditions, as described previously [24,27,28,29]. In short, α-epoxyarglabin (**1a**) and β-epoxyarglabin (**1b**) were obtained by epoxidation of arglabin (**1**) with peracetic acid [27]. 1β,10α-Epoxyargolide (**2a**) was obtained by epoxidation of argolide (**2**) with trifluoroperacetic acid [28]. Epoxyarhaline (**4a**) was synthesized via epoxidation of 3β-hydroxyarhalin (**4**) by *m*-chloroperbenzoic acid [24]. Cytisine or anabasine moieties were introduced into compounds **1**, **1b**, **2**, and **3** via Michael amination, as reported previously [29]. The reaction products were compounds **1d**, **2b**, and **3b** for cytisine and compounds **1e** and **1f** for anabasine derivatives. Their proton magnetic resonance (^1^H-NMR) spectra lacked resonances for the exomethylene protons, indicating that the relevant alkaloid (cytisine or anabasine) was added to the exomethylene double bond of the γ-lactone moiety. Cytisine or anabasine moieties were introduced into the methylene motif of arglabin, epoxyarglabin, and grosheimin to enrich the chemical diversity. It was concluded that cytisine had an α-orientation and the anabasine moiety had a β-configuration [29]. Pyridinyl arglabin (**1c**) was synthesized by a reaction of arglabin (**1**) with 3-iodopyridine. Chloroacetate grosheimin (**3a**) was synthesized by a reaction of grosheimin (**3**) with chloroacetic anhydride. The full details of the synthesis of pyridinyl arglabin and chloroacetate grosheimin will be reported elsewhere.

The structures of the natural SLs and their semi-synthetic derivatives were elucidated using spectral data (IR, ^1^H-NMR, ^13^C-NMR, DEPT, COSY), mass spectrometry, elemental analysis, and X-ray crystal structure analysis, in addition to comparisons with the literature. The structures of the compounds under investigation are shown in Figure 1.

### 2.2. Effects of Semi-Synthetic SL Derivatives on TCR Activation

**Figure 2:**
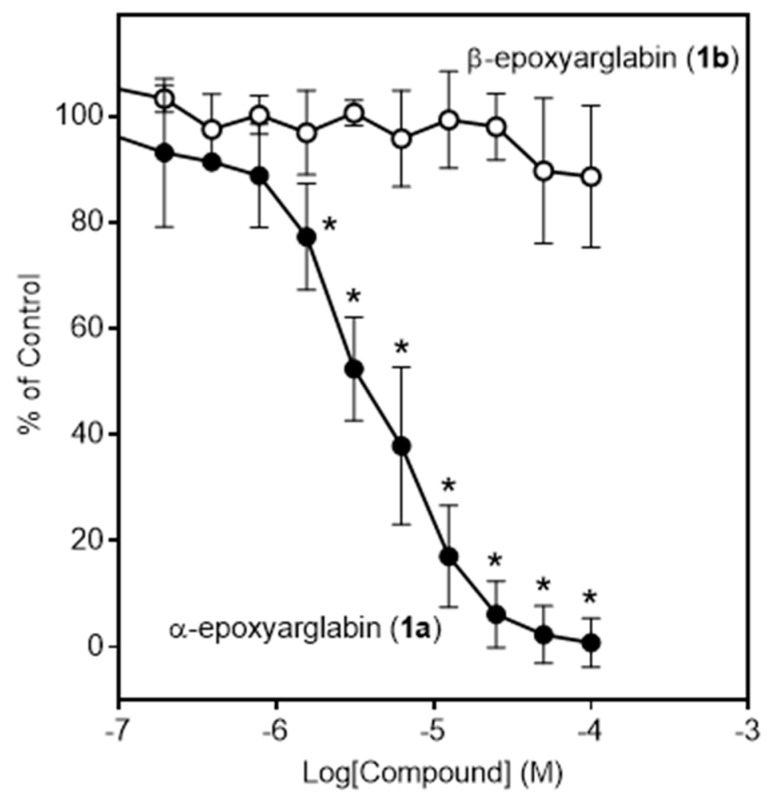
Effect of α-epoxyarglabin (**1a**) and β-epoxyarglabin (**1b**) on activation-induced Ca^2+^ mobilization in Jurkat T cells. Jurkat T cells were pretreated for 20 min with a control of 1% DMSO or increasing concentrations of the indicated compounds and activated with anti-CD3. Activation-induced Ca^2+^ flux was measured as described, and the results are shown as the % of maximal activation measured in the control cells. The results shown in both panels are representative of three independent experiments. Statistically significant differences between cells pretreated with compound **1a** versus compound **1b** are indicated (* *p* < 0.01).

To assess the biological activity of the 11 semi-synthetic SL analogs and a natural analog of argolide (dihydroargolide or ketopelenolide B), we evaluated their effects on TCR activation-induced responses in Jurkat T cells and compared these effects with the previously measured responses of the parent compounds **1**–**4** [6]. TCR activation by anti-CD3 antibodies resulted in the rapid mobilization of intracellular Ca^2+^ [30,31]. As shown in Table 1, α-epoxyarglabin (**1a**), cytisinyl epoxyarglabin (**1d**), 1β,10α-epoxyargolide (**2a**), and chloroacetate grosheimin (**3a**) all dose-dependently inhibited anti-CD3-induced intracellular Ca^2+^ flux with IC_50_ values in the micromolar range, whereas the other analogs were inactive. As an example, a representative dose–response curve for the inhibition of Jurkat T cell Ca^2+^ mobilization by α-epoxyarglabin is shown in Figure 2. Notably, in this assay the semi-synthetic analogs appear to be more potent than the parent compounds. It is also interesting that β-epoxyarglabin (**1b**), the stereoisomer of α-epoxyarglabin (**1a**), was completely inactive (Figure 2), suggesting a stereo-specific effect.

To consider whether the active SLs also inhibited Ca^2+^ flux in other phagocytes or whether this effect was specific for T cells, we evaluated whether the compounds that were active in Jurkat T cells could alter the chemotactic peptide-induced Ca^2+^ flux in human neutrophils or HL60 cells transfected with *N*-formyl peptide receptor 2 (FPR2-HL60). Treatment of these cells for 20 min with α-epoxyarglabin (**1a**), cytisinyl epoxyarglabin (**1d**), or 1β,10α-epoxyargolide (**2a**) had no effect on the Ca^2+^ mobilization induced by *N*-formylmethionyl-leucyl-phenylalanine (*f*MLF) in neutrophils or hexapeptide WKYMVM in FPR2-HL60 cells (Table 1). In contrast, chloroacetate grosheimin (**3a**) inhibited Ca^2+^ flux in FPR2-HL60 cells and human neutrophils, indicating that its inhibitory effects were not specific to TCR activation. Indeed, subsequent experiments showed that compound **3a** was actually cytotoxic for Jurkat T cells, while the other active analogs had no cytotoxic activity (Table 1). 

ERK1/2 phosphorylation is one of the main TCR activation-induced responses [32]. Thus, we also evaluated effects of the SL derivatives on this response. Although none of the compounds directly stimulated ERK1/2 phosphorylation (data not shown), pretreatment of Jurkat T cells with various concentrations of the test compounds, followed by activation with anti-CD3/CD28 antibodies, showed that the four analogs that inhibited Ca^2+^ mobilization (**1a**, **1d**, **2a**, and **3a**) also significantly inhibited TCR activation-induced ERK1/2 phosphorylation in a dose-dependent manner (Table 1). Similar to the Ca^2+^ flux assay, compound **2b** was inactive in the ERK1/2 phosphorylation assay.

### 2.3. Glutathione (GSH) Reactivity of Semi-Synthetic SL Derivatives

The TCR-mediated response is sensitive to changes in intercellular GSH levels ([GSH]_i_) [33]. Some SLs, including the parent compounds **1**–**4**, can decrease [GSH]_i_ in various human cells [6,34,35]. We evaluated if the SL derivatives reacted with GSH in Jurkat T cells and found that the derivatives which inhibited Ca^2+^ mobilization and ERK1/2 phosphorylation also depleted [GSH]_i_ in Jurkat T cells (Table 1). Although chloroacetate grosheimin (**3a**) also depleted [GSH]_i_, its cytotoxicity and lack of specificity (see above) precluded it from being a useful inhibitor. While α-epoxyarglabin (**1b**) was one of the most potent compounds in the Ca^2+^ flux assay, its ability to deplete [GSH]_i_ in Jurkat cells was lower than that of the active compounds. 

### 2.4. Molecular Modeling 

**Scheme 1:**
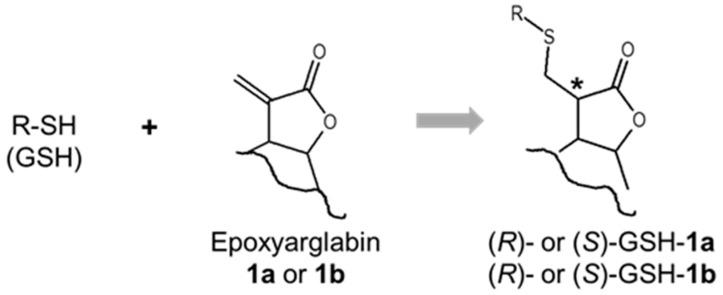
Model reaction between GSH and α- and β-epoxyarglabines in aqueous medium. The chiral center is marked by the asterisk.

The interaction of SLs containing an α,β-unsaturated moiety with the GSH cysteine residue is accompanied by the nucleophilic addition of the SH-group to the carbon–carbon double bond [36,37]. To estimate if the geometry of stereoisomeric SLs affects their interaction with GSH, we performed a quantum-chemical investigation of the model reaction between GSH and α- and β-epoxyarglabines in an aqueous medium (Scheme 1) using the density functional theory (DFT) method.

**Figure 3:**
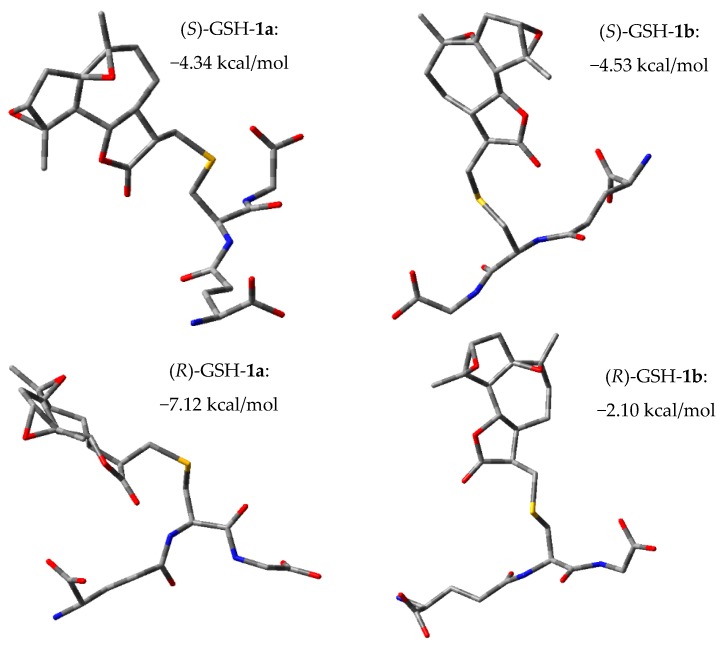
Molecular conformations and changes in Gibbs free energy calculated by the DFT method for the reaction products according to Scheme 1.

The addition of compound **1a** or **1b** to GSH led to the appearance of an additional chiral center (see asterisk in Scheme 1). Thus, the DFT calculations of the reaction thermodynamics were performed for both pairs of possible diastereomeric products. GSH is conformationally flexible, hence we started our calculations using the geometry of GSH close to its extended conformation, which was recently determined by spectroscopic methods and is considered to be biologically important [38]. Geometric optimization followed by vibrational frequency calculations for the reaction shown in Scheme 1 resulted in the computed changes in Gibbs free energy ΔG^o^, as summarized in Figure 3.

The formation of all four isomers is thermodynamically favorable, as indicated by the negative values of ΔG^o^ for the nucleophilic addition reaction. In absolute values, the changes in Gibbs energy were not large. However, one would not expect extreme negative values under mild intracellular conditions. A comparison of ΔG^o^ magnitudes showed that for β-epoxyarglabin (**1b**) the addition product would be formed predominantly with an *S*-configuration of the newly arising chiral center, whereas for α-epoxyarglabin (**1a**) a diastereomer with the *R*-configuration would predominantly be formed. In addition, the formation of (*R*)-GSH-**1a** is the most thermodynamically favorable, as the reaction is characterized by the most negative ΔG^o^ value (Figure 3). This finding is consistent with the biological activity observed for α-epoxyarglabin (**1a**), which was not evident for its stereoisomer β-epoxyarglabin (**1b**). It should be noted that compounds **1a** and **1b** can be regarded as diastereomers. Hence, their properties, including their reaction thermodynamics and affinity to a biotarget, are expectedly different, as is known for diastereomeric pairs [39,40]. A comparison of the ΔG^o^ values obtained for nucleophilic addition (Scheme 1 and Figure 3), where significantly higher changes in Gibbs free energy were calculated for the interaction of GSH with the OH radical [41], is in agreement with the mild character of the S–H bond reaction with the α-methylene moiety of SLs. 

While α-epoxyarglabin (**1a**) effectively inhibited T cell activation, it had little effect on [GSH]_i_ and only depleted Jurkat cell GSH by 30% at the highest concentrations tested (50–100 μM/L). Indeed, it was recently found that GSH is dispensable for early T cell activation [42]. Thus, the specific reaction of SLs with cysteine residues of target proteins may also involve the shape of the compounds and the ability to interact with a specific binding site [37,43,44]. For example, parthenolide has been found to covalently target Cys38 of NF-κB p65 through hetero-Michael addition between exocyclic methylene butyrolactones and the SH-group of Cys38 [45].

Upon TCR activation, ZAP-70 is recruited to the TCR complex, where it is activated and co-localized to its substrates [46]. This kinase contains nine cysteine residues in the tandem Src homology 2 (SH2) module and seven cysteine residues in the kinase domain. Several small-molecule compounds that reacted covalently with the cysteine residues of this module and inhibited ZAP-70 binding to phosphorylated immune-receptor tyrosine-based activation motif (ITAM)-derived peptides were recently identified [2,47]. Because the SLs identified here have the potential to conjugate with cysteine residues on target molecules and the ZAP-70 SH2 region is enriched in cysteine residues, we used molecular modeling to assess the potential interaction of these SLs with the ZAP-70 tandem-SH2 module using the three-dimensional structures of these natural compounds.

For molecular modeling studies, the terminal carbon atom of the SL α-methylene moiety (=CH_2_) and the sulfur of cysteine residues in the ZAP-70 SH2 module were specified as reactive ligands and target atoms (reaction centers). Of the nine cysteine residues in the tandem-SH2 module of ZAP-70, five are in the N-SH2 domain (Cys39, Cys78, Cys84, Cys96 and Cys102); Cys117 is located in the inter-SH2 domain linker and three others (Cys222, Cys249 and Cys254) are located in the C-SH2 domain [47]. Of these targets, molecular docking was conducted for Cys39, Cys78, Cys96, Cys102, Cys222, Cys249, and Cys254, and we found that stable poses with negative docking score values were obtained only for binding sites around Cys39 and Cys78. Taking into account the importance of Cys39 in biological activity, we focused on the docking results of the cavity containing this cysteine. Indeed, covalent adducts between small-molecule inhibitors of the ZAP-70-TCR association were found only for Cys39 and Cys78 [47]. The distances C…S between the proposed reaction centers mentioned above were calculated for the docking poses obtained (Table 2). Lower values of d(C…S), falling between 3.04–5.25 Å in the vicinity of Cys39, corresponded to the active SLs, including argracin, estafiatine, and parthenolide. The distances in this range are favorable for nucleophilic addition of the Cys39 S–H bond to the exocyclic double bond of the lactone ring. 

**Figure 4:**
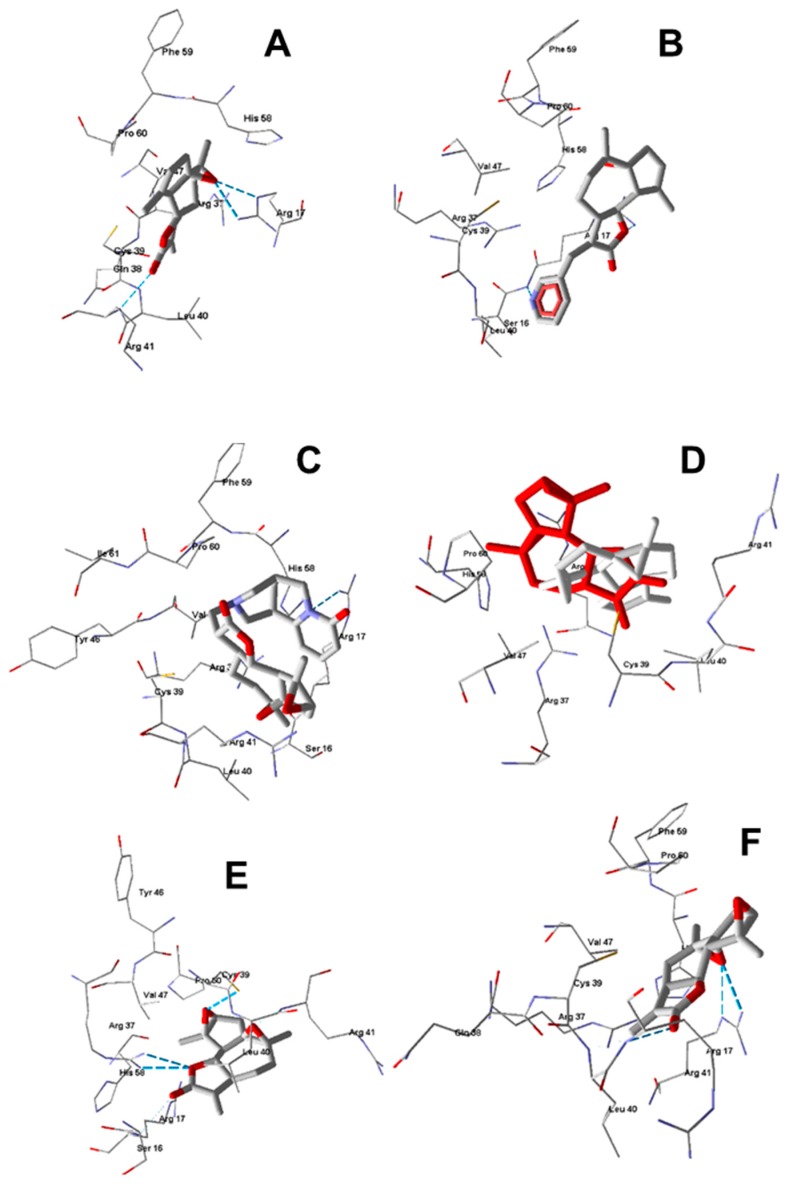
Docking poses of arglabin and its semi-synthetic derivatives in the ZAP-70 SH2 module. Panel (**A**) Docking pose of arglabin (**1**). Panel (**B**) Docking pose of pyridinyl arglabin (**1c**). Panel (**C**) Docking pose of cytisinyl epoxyarglabin (**1d**). Panel (**D**) Superposition of docking poses of α-epoxyarglabin (**1a**; red) and β-epoxyarglabin (**1b**; grey). Panel (**E**) Docking pose of β-epoxyarglabin (**1b**). Panel (**F**) Docking pose of α-epoxyarglabin (**1a**). In Panel D, residues within 7 Å of the center of the search space are shown. In all other panels, residues within 4 Å of the pose are shown. H-bonds are shown as blue dashes.

Arglabin (**1**) forms an H-bond with Arg41 of the ZAP-70 SH2 module through participation of the carbonyl oxygen atom and is strongly H-bonded to Arg17 via the epoxide oxygen atom (Figure 4A). The molecule also has significant non-bonded attraction to Pro60, Val47, Leu40, and Arg37 in close proximity to the docking pose. The docking pose of pyridinyl arglabin (**1c**) is shown in Figure 4B. As expected, the bulky pyridine ring had a significant effect on the molecule’s orientation compared to that of arglabin (**1**). It forms H-bonds with two different nitrogen atoms of Arg17, with participation of the pyridine nitrogen and lactone oxygen atoms (Figure 4B). As a result of this fixation, the exocyclic C=C bond in the lactone moiety appears to be in an unfavorable orientation with respect to the Cys39 SH-group. Moreover, electronic conjugation with the pyridine heterocycle makes the reaction center less prone to nucleophilic attack by the sulfur atom of Cys39. In contrast, cytisinyl epoxyarglabin (**1d**) does not possess an exocyclic double bond and is characterized by the most advantageous docking score (−104 kcal/mol) (Table 2). For most of the other compounds investigated, the docking scores did not exceed 85 kcal/mol in absolute values. It is possible that a greater ability to be retained inside the kinase cavity led to the high biological activity of **1d**. It is interesting that this high docking score is mainly due to the van der Waals attraction, because the molecule forms only one H-bond of an intermediate strength with Arg17 (Figure 4C). In comparison, inactive anabasinyl epoxyarglabin (**1e**) does not possess an exocyclic C=C bond in the lactone moiety and has a substantially lower docking score in absolute values (−85 kcal/mol) than its active analogue **1d**.

In Figure 4D, the docking poses of α-epoxyarglabin (**1a**) and β-epoxyarglabin (**1b**) are shown for comparative purposes. The different arrangements of these isomers within the active site are apparently due to the dissimilar orientations of the epoxide cycles. The inactive β-epoxy isomer **1b** forms a strong H-bond with Cys39 (Figure 4E). Additionally, H-bonds of the lactone cycle with Arg37 are formed. Thus, Cys39 cannot nucleophilically attack the terminal CH_2_ group. The active α-epoxy isomer **1a** does not form H-bonds with help from the α-epoxy cycle (Figure 4F), but it forms H-bonds with Arg17 through another epoxide and with Arg41 through the carbonyl group. With this orientation, an attack by the sulfur atom of Cys39 on the terminal CH_2_ group is possible.

**Figure 5:**
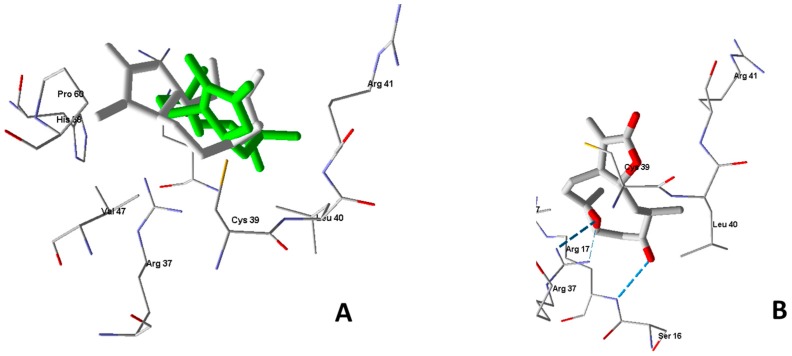
Docking poses of argolide (**2**) and 1β,10α-epoxyargolide (**2a**) and in the ZAP-70 SH2 module. Panel (**A**) is the docking poses of 1β,10α-epoxyargolide (**2a**) (green) and argolide (**2**) (grey). Residues within 7 Å of the center of the search space are shown. Panel (**B**) is the docking pose of 1β,10α-epoxyargolide (**2a**). Residues within 3 Å of the pose are shown. H-bonds are shown as blue dashes.

For argolide (**2**), the d(C…S) value is about 6.5 Å and should be considered too long for an effective nucleophilic attack of the S center to the terminal olefin carbon atom. This result is consistent with the lack of biological activity of compound **2**. Although argolide docking shows a good distance between its exocyclic double bond and the S–H bond of Cys78 (3.69 Å), adduct formation at this residue does not block SH2 module binding with ITAM [47]. A comparison of the docking results for active 1β,10α-epoxyargolide (**2a**) and its inactive precursor argolide (**2**) shows that they have quite differently arranged scaffolds within the binding site in the vicinity of Cys39 (Figure 5A). In Figure 5B, the pose of the semi-synthetic derivative **2a** is shown separately with the amino acid residues within 3 Å of this pose. The molecule is fixed in the binding site by strong H-bonds with Arg17 and Arg37. The latter of these H-bonds is formed with participation of the epoxy oxygen, i.e., the possibility of its formation is due to the chemical modification of argolide. As a result of this fixation, the terminal CH_2_ group appears to be favorably oriented with respect to the SH-group of Cys39. Natural and semi-synthetic argolide derivatives, dihydroargolide (**2c**), and cytisinyl argolide (**2b**) have no possibility for nucleophilic addition to the cysteine SH group. Also, they are characterized by relatively low docking scores (Table 2), which is consistent with their inability to inhibit TCR activation. 

**Figure 6:**
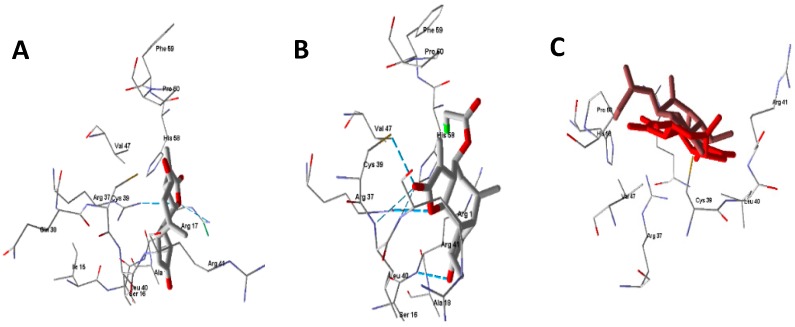
Docking poses of grosheimin (**3**) and chloroacetate grosheimin (**3a**) in the ZAP-70 SH2 module. Panel (**A**). Docking pose of grosheimin (**3**). Panel (**B**). Docking pose of chloroacetate grosheimin (**3a**). Panel (**C**). Superimposed docking poses of the grosheimin (**3**; red) and chloroacetate grosheimin (**3a**; brown). In each panel, residues within 4 Å of the pose are shown. H-bonds are shown as blue dashes.

Moderately active grosheimin (**3**) is characterized by a higher d(C…S) value at Cys39 than the other active compounds (see Table 1 and Table 2). Grosheimin (**3**) forms H-bonds of its lactone oxygen atom with Arg37 and Arg17 (Figure 6A). Nevertheless, the distance from the Cys39 sulfur atom to the =CH_2_ group can be considered quite acceptable. In the pose of compound **3a**, H-bonds are formed with Cys39, Arg37, and Arg17 and involve the participation of both oxygen atoms of the lactone cycle (Figure 6B). Weaker H-bonds are also formed with Leu40 and Arg41. A comparison of the docking poses of **3** and **3a** is shown in Figure 6C. 3β-Hydroxyarhaline (**4**) and epoxyarhaline (**4a**) belong to the group of compounds without activated double bonds and have relatively low absolute docking scores (Table 2). Their lack of biological activity is consistent with the observations described above regarding the lower absolute value of their docking scores compared to that of active cytisinyl epoxyarglabin (**1d**).

We also performed a docking studies of the previously reported active natural SLs argracin, estafiatine, and parthenolide [6]. The results for these compounds were consistent with the described conditions required for biological activity, i.e., these molecules contained an exocyclic C=C bond activated by the neighborhood of a carbonyl group. Additionally, the geometric features of the docking poses are favorable for nucleophilic addition of the Cys39 SH-group (i.e., the C…S distances are relatively short and lie between 4.12 and 4.70 Å) (Table 2). In general, our molecular docking results showed that the SLs and their semi-synthetic derivatives could be differentiated by geometric features in the receptor cavity near Cys39, which impacted their ability to react with the protein SH-group.

We evaluated the immunomodulatory effects of 11 semi-synthetic derivatives of SLs and dihydroargolide and found that four of the tested compounds [α-epoxyarglabin (**1a**), cytisinyl epoxyarglabin (**1d**), 1β,10α-epoxyargolide (**2a**), and chloroacetate grosheimin (**3a**)] inhibited the early phases of TCR activation. The activation of Jurkat T cells was suppressed by these compounds, as demonstrated by the inhibition of Ca^2+^ mobilization and ERK1/2 phosphorylation. The active compounds also depleted [GSH]_i_. Although several natural SLs were previously reported to modulate T cell activity stimulated through the TCR [6], this is the first report demonstrating inhibition of TCR activation-induced Ca^2+^ mobilization and ERK1/2 phosphorylation by semi-synthetic SL derivatives. The precise target of the active SLs and their derivatives is not currently known, although we, along with others, reported that the α-methylene-γ-lactone group is important for their biological activity [6,48,49]. Indeed, substitution of the methylene motif led to inactive anabasinyl epoxyarglabin (**1e**), anabasinyl arglabin (**1f**), cytisinyl argolide (**2b**), and cytisinyl grosheimin (**3b**). These results further demonstrate the importance of the α-methylene functionality. Although the differences in activity between individual SLs may be explained by their different reactive moieties (e.g., α,β-unsaturated bond, epoxide group, etc.) [6,48,49], our results also suggested some specificity the in biological action of semi-synthetic SLs bearing equal numbers of these reactive moieties. For example, α-epoxyarglabin (**1a**), but not its stereoisomer β-epoxyarglabin (**1b**), inhibited TCR activation.

It was reported recently that GSH is dispensable for initial T cell activation [42]. On the other hand, α-epoxyarglabin (**1a**) had only a minimal effect on GSH levels (an order of magnitude weaker than that of other active SLs and their derivatives). Therefore, while the results of our computations correspond to the ratios in activity for these compounds, we considered that molecular targets other than GSH may exist in the cell and that SLs may also directly react with proteins [50]. It is clearly feasible that the semi-synthetic SL derivatives could form adducts with macromolecules engaged in TCR-dependent activation. Upon TCR stimulation, tyrosine kinases Fyn and Lck phosphorylate ITAMs in the TCR recruit tyrosine kinases Syk and ZAP-70 to phosphorylate the linker of activated T cells (LAT), which results in the formation of a complex consisting of various components, including interleukin-2-inducible tyrosine kinase (Itk) and phospholipase C (PLC) γ1 [51,52]. Using a high-throughput screen, Visperas et al. [2,47] recently identified several small-molecule compounds that covalently reacted with cysteine residues of the ZAP-70 tandem SH2 module and inhibited its binding to a phosphorylated ITAM-derived peptide. Thus, based on the reactivity of the active SLs and their derivatives for cysteine, it is possible that they can similarly induce redox-dependent post-translational modification of cysteine residues in the ZAP-70 SH2 module to regulate its function. Here, we conducted molecular docking to elucidate possible binding modes between the ZAP-70 tandem SH2 module and the SLs, including their semi-synthetic derivatives. 

Previously, docking experiments were conducted to predict the formation of covalent protein adducts of parthenolide and semi-synthetic SL derivatives with Cys38 located in the p65 subunit of NF-κB [20,53]. Another recent docking study showed that parthenolide and its derivatives interact with critical amino acid residues of inhibitor of NF-κB kinase subunit β (IKKβ) in a non-covalent fashion [54]. In our docking experiments, we focused on the receptor cavity in the vicinity of Cys39, which was determined to be important for ligand-receptor interactions with ZAP-70 [2,47]. Our docking results showed that for biologically active compounds, the exocyclic C=C bond in the lactone moiety is located near the SH-group of Cys39 and can be subject to nucleophilic attack by the sulfur atom. Indeed, the distances d(C…S) between the terminal carbon and the Cys39 sulfur atom were all less than 6 Å for active SLs. The ligand positions within the binding site were determined by H-bonding interactions. However, in the case of compound **1d**, the high absolute value of the docking score was due to the Van-der-Waals interactions with ZAP-70. This may be the reason for the biological activity of compound **1d** despite the absence of the activated C=C bond.

Overall, these results suggest a potential new strategy for developing novel therapeutics based on natural SLs that could effectively modulate TCR responses. Further detailed studies are warranted to define the molecular targets and the therapeutic potential of these natural SLs as previously undescribed immunomodulatory and anti-inflammatory agents.

## 3. Materials and Methods

### 3.1. Plant Material and Isolation of Natural SLs

Arglabin, argolide, 3β-hydroxyarhalin, dihydroargolide, and grosheimin were isolated from different plants of the *Asteraceae* family, as previously described [23,24,25,26,55]. The purity of each compound was determined to be >98% by a normalization of the peak areas detected on an automated high-performance liquid chromatography (HPLC) system (Hewlett–Packard Agilent 1100, Santa Clara, CA, USA) with a Zorbax SB-C_18_ column (4.6 × 150 mm), eluted with acetonitrile/water (50%/50%, *v*/*v*) or methanol/water (50%/50%, *v*/*v*) at a flow rate of 0.5 mL/min at 25 °C. The elution was monitored at 204 nm.

### 3.2. Synthesis of Derivatives from Natural SLs

Derivatives of the isolated SLs (see Figure 1 for the structures), with the exception of chloroacetate grosheimin and pyridinyl arglabin, were synthesized using optimized reaction conditions, as described previously [24,27,28,29]. The structures of the natural SLs and their semi-synthetic derivatives were elucidated using spectral data (IR, UV, ^1^H-PMR, ^13^C-NMR, DEPT, COSY), mass spectrometry, elemental analysis, and X-ray crystal structure analysis, in addition to comparisons with the literature.

### 3.3. Materials for Biological Assays

Dimethyl sulfoxide (DMSO), N-formyl-methionine-leucine-phenylalanine (*f*MLF), and Histopaque 1077 were purchased from Sigma-Aldrich Chemical Co. (St. Louis, MO, USA). The Fluo-4AM dye was from Invitrogen (Carlsbad, CA, USA). Anti-human CD3 and anti-human CD28 monoclonal antibodies were purchased from eBioscience (San Diego, CA, USA). The Ficoll-Paque was from GE Healthcare Bio-Science AB (Uppsala, Sweden). The penicillin–streptomycin solution was purchased from Mediatech (Herndon, VA, USA). Fetal bovine serum (FBS) was purchased from Atlas Biologicals (Fort Collins, CO, USA). The Hanks’ balanced salt solution (HBSS; 0.137 M NaCl, 5.4 mM KCl, 0.25 mM Na_2_HPO_4_, 0.44 mM KH_2_PO_4_, 4.2 mM NaHCO_3_, 5.56 mM glucose, and 10 mM HEPES, pH 7.4) was from Life Technologies (Grand Island, NY, USA). HBSS without Ca^2+^ and Mg^2+^ was designated as HBSS^−^; HBSS containing 1.3 mM CaCl_2_ and 1.0 mM MgSO_4_ was designated as HBSS^+^.

### 3.4. Cell Culture

Human Jurkat T acute lymphoblastic leukemia cells and human promyelocytic leukemia cells (HL60 cells) stably transfected with FPR2 (FPR2-HL60 cells) were cultured in RPMI-1640 (Mediatech, Inc., Herndon, VA, USA) supplemented with 10% heat-inactivated FBS, 10 mM HEPES, 100 μg/mL streptomycin, and 100 U/mL penicillin. Transfected HL60 cells were cultured in the presence of G418 (1 mg/mL). All cell lines were incubated in a humidified incubator at 37 °C with an atmosphere of 5% CO_2_.

### 3.5. Isolation of Human Neutrophils

For the isolation of human neutrophils, blood was collected from healthy donors in accordance with a protocol approved by the Institutional Review Board at Montana State University (Protocol #MQ041017). The neutrophils were purified from the blood using dextran sedimentation, followed by Ficoll-Paque 1077 gradient separation and hypotonic lysis of the red blood cells, as described previously [56]. Isolated neutrophils were washed twice and resuspended in HBSS. Neutrophil preparations were routinely >95% pure, as determined by light microscopy, and >98% viable, as determined by trypan blue exclusion. Neutrophils were obtained from multiple donors (*n* = 8); however, the cells from different donors were never pooled together during experiments.

### 3.6. ERK1/2 Enzyme-Linked Immunosorbent Assay (ELISA)

Jurkat T cells were incubated for 20 min with the selected compounds or negative control (1% DMSO) at 37 °C, followed by addition of anti-CD3/C28 monoclonal antibodies (10 µg/mL of each antibody) and a 5-min incubation at room temperature. The cells were lysed with lysis buffer (R&D Systems, Minneapolis, MN, USA), and the levels of phosphorylated ERK1/2 were measured in the cell lysates using an ELISA kit (R&D Systems) for human phospho-ERK1 (Thr202/Tyr204)/ERK2 (Thr185/Tyr187). The concentrations of phospho-ERK1/2 in the cell lysates were determined using a calibration curve with recombinant human phospho-ERK2 (Thr85/Tyr187).

### 3.7. Ca^2+^ Mobilization Assay

Changes in intracellular Ca^2+^ concentrations ([Ca^2+^]_i_) were measured with a FlexStation 3 scanning fluorometer (Molecular Devices, Sunnyvale, CA, USA). Briefly, cells (human neutrophils, FPR2-HL60 cells or Jurkat cells) were suspended in HBSS, loaded with Fluo-4AM at a final concentration of 1.25 μg/mL and incubated for 30 min in the dark at 37 °C. After dye loading, the cells were washed with HBSS^−^, resuspended in HBSS^+^, separated into aliquots, and aliquoted into the wells of flat-bottom, half-area well black microtiter plates (2 × 10^5^ cells/well). Test compounds diluted in DMSO were added to the wells (the final concentration of DMSO was 1%), and changes in fluorescence were monitored (λ_ex_ = 485 nm, λ_em_ = 538 nm) every 5 s for 240 s at room temperature after addition of test compounds to evaluate direct agonist effects. To evaluate inhibitory effects, Jurkat cells were pretreated for 20 min with various concentrations of test compounds, followed by the addition of 10 µg/mL anti-CD3 antibody. The maximum change in fluorescence, expressed in arbitrary units over baseline, was used to determine the agonist response. Responses were normalized to the response induced by anti-CD3 for Jurkat cells, 5 nM *f*MLF (neutrophils), or 5 nM WKYMVM (FPR2-HL60 cells), which was assigned a value of 100%. Curve fitting (at least five or six points) and calculation of median effective concentration values (IC_50_) were performed by nonlinear regression analysis of the dose–response curves generated using Prism 7 (GraphPad Software, Inc., San Diego, CA, USA).

### 3.8. Assessment of Compound Cytotoxicity

Cytotoxicity was analyzed with a CellTiter-Glo Luminescent Cell Viability Assay Kit (Promega, Madison, WI, USA). Briefly, Jurkat T cells were cultured at a density of 2 × 10^5^ cells/well with different concentrations of the compounds under investigation for 30 min at 37 °C. Following treatment, substrate was added, and the samples were analyzed with a Fluoroscan Ascent FL microplate reader (Thermo Electron, Waltham, MA, USA).

### 3.9. Glutathione (GSH) Assay

Jurkat T cells were plated at 10,000 cells per well in white 96-well half-area plates. After addition of test SLs or a vehicle (1% DMSO) negative control, the cells were incubated for 30 min at 37 °C, and total GSH was measured using a GSHGlo™ assay (Promega Corp., Madison, WI, USA), according to the manufacturer’s instructions [57]. Luminescence was measured with a Fluroscan Ascent FL microplate reader.

### 3.10. DFT Calculations

The molecular models of compounds **1a** and **1b** were built using ChemDraw 16.0 software (PerkinElmer, Waltham, MA, USA) and refined by molecular mechanics with the MM2 force field. Similarly, 3D models of all other investigated SLs and their semi-synthetic derivatives were prepared for the docking studies (see below). 

The 3D conformer of GSH was downloaded from the PubChem website [58] and imported into ChemDraw 16.0, where its geometry was modified to the extended conformation [38] with manual editing of the torsion angles. To obtain the initial geometries of the adducts GSH-**1a** and GSH-**1b** in their *R*- and *S*- configurations (Scheme 1), we attached the 3D models of compounds **1a** or **1b** to the sulfur atom instead of the hydrogen atom in the extended conformation of GSH using HyperChem 7 software (Hypercube, Inc., Gainesville, FL, USA). Torsion angles at the junction of GSH and sesquiterpene moieties in initial structures of the adducts were set as follows: 60° (N-C-C-S), 180° (C-C-S-C^β^), 60° (C-S-C^β^-C^α^), 60° (S-C^β^-C^α^-C^carbonyl^, *R*-configuration), and −60° (S-C^β^-C^α^-C^carbonyl^, *S*-configuration), where C^carbonyl^, C^α^, and C^β^ are the corresponding atoms of the substituted lactone moiety. The chosen values of the initial torsion angles allowed us to avoid steric clashes between GSH and the sesquiterpene parts of the adducts.

All reactants and products from Scheme 1 were pre-optimized by the PM6 method using a Gaussian 16 package [59]. Subsequently, geometry optimization and frequency calculation were performed in Gaussian 16 by the DFT method with B3LYP functional [60,61] and 6-31+G(d,p) basis sets. The solvent (water) was accounted for using the PCM solvation model. Real energy minima with no imaginary frequencies were attained for each reaction participant. For the optimized geometries, high-quality single point runs were made with the M11 functional [62] and 6-311++G(2d,2p) basis sets. Gibbs free energies were calculated from electronic energies obtained at the M11/6-311++G(2d,2p) level of theory with zero-point corrections from the B3LYP/6-31+G(d,p) approximation. All Gaussian 16 jobs were performed on a 24-core 3 MHz server operating under the Ubuntu 16.04 system (Canonical, Ltd., London, UK).

### 3.11. Molecular Docking

The molecular model of ZAP-70 was downloaded from the Protein Data Bank (2OZO entry) and imported into the Molegro Virtual Docker (MVD) 6.0 program (Molegro ApS, Aarhus, Denmark). All water molecules were removed. In the vicinity of each cysteine residue of the SH2 module (Cys39, Cys78, Cys96, Cys102, Cys222, Cys249, and Cys254), a spherical search space was defined which embraced the cysteine and other nearest residues that formed the cavity of the putative binding site. The radius of the sphere was 7 Å for Cys39 and 8 Å for other cavities. All residues within the cavities were considered flexible; a softening parameter of 0.7 was applied during flexible docking with MVD.

The structures of the compounds, prepared as described above, were imported into the MVD program, and 30 docking runs were performed for each molecule in each of the seven potential binding sites with the default options of MVD. The docking poses that obtained negative docking scores were saved, and these low-energy poses were analyzed with the measurement tools of MVD.

## Figures and Tables

**Table 1:** Effect of SLs on Ca^2+^ mobilization and ERK1/2 phosphorylation in Jurkat T cells.

Name (Compound Number)	Jurkat T Cells		FPR2-HL60	PMN	Jurkat T Cells	Cyto-Toxicity
	Ca^2+^ Flux	pERK1/2	Ca^2+^ flux		[GSH]_i_	
	IC_50_ (μM)					
Arglabin (**1**) *	11.1 ± 2.7	28.7 ± 6.3	N.A.	N.A.	18.0 ± 1.8	N.T.
α-Epoxyarglabin (**1a**)	4.4 ± 1.8	32.1 ± 11.5	N.A.	N.A.	(30%)	N.T.
β-Epoxyarglabin (**1b**)	N.A.	N.A.	N.A.	N.A.	N.A.	N.T.
Pyridinyl arglabin (**1c**)	N.A.	N.A.	N.A.	N.A.	N.A.	N.T.
Cytisinyl epoxyarglabin (**1d**)	3.9 ± 1.2	15.4 ± 3.3	N.A.	N.A.	12.4 ± 3.5	N.T.
Anabasinyl epoxyarglabin (**1e**)	N.A.	N.A.	N.A.	N.A.	N.A.	N.T.
Anabasinyl arglabin (**1f**)	N.A.	N.A.	N.A.	N.A.	N.A.	N.T.
Argolide (**2**) *	N.A.	N.A.	N.A.	N.A.	N.A.	N.T.
1β,10α-Epoxyargolide (**2a**)	14.0 ± 3.4	39.4 ± 8.5	N.A.	N.A.	36.1 ± 9.4	N.T.
Cytisinyl argolide (**2b**)	N.A.	N.A.	N.A.	N.A.	N.A.	N.T.
Dihydroargolide (**2c**)	N.A.	N.A.	N.A.	N.A.	N.A.	N.T.
Grosheimin (**3**) *	15.4 ± 4.3	43.0 ± 7.5	N.A.	N.A.	16.6 ± 4.8	N.T.
Chloroacetate grosheimin (**3a**)	6.0 ± 2.1	7.8 ± 2.9	13.2 ± 3.4	10.1 ± 2.8	3.8 ± 1.4	12.3 ± 3.6
Cytisinyl grosheimin (**3b**)	N.A.	N.A.	N.A.	N.A.	N.A.	N.T.
3β-Hydroxyarhaline (**4**) *	N.A.	N.A.	N.A.	N.A.	N.A.	N.T.
Epoxyarhaline (**4a**)	N.A.	N.A.	N.A.	N.A.	N.A.	N.T.

Inhibitory activity was evaluated after a 20 min pretreatment with various concentrations of the indicated compounds at room temperature, followed by the addition of 10 µg/mL anti-CD3/CD28 (pERK1/2 in Jurkat cells), 10 µg/mL anti-CD3 (Ca^2+^ flux in Jurkat cells), 5 nM *f*MLF [Ca^2+^ flux in human neutrophils (PMN)], or 5 nM WKYMVM (Ca^2+^ flux in FPR2-HL60 cells). N.A. (no activity) or N.T. (no toxicity) was observed at all concentrations tested (5.125, 6.25, 25, and 50 µM). Percent inhibition is indicated at 50 µM of SLs if full inhibition was not observed at the highest tested concentration (50 µM) in the GSH assay. * Activities for parent compounds were taken from Reference [6].

**Table 2:** Distances between the S–H bond in the cysteine residues of the ZAP-70 tandem-SH2 module and the exocyclic double bond of the lactone ring in the SLs predicted by molecular docking.

Compound	Name	d(C…S), Å		Docking Score (Cys39 Site)	Activity (Inhibition of TCR Response)
		*Cys39*	*Cys78*		
**1**	Arglabin	**3.55**	6.74	−67.6	Active ^a^
**1a**	α-Epoxyarglabin	**3.77**	5.36	−72.5	Active
**1b**	β-Epoxyarglabin	7.35	5.18	−78.6	N.A.
**1c**	Pyridinyl arglabin	6.15	6.85	−60.9	N.A.
**1d**	Cytisinyl epoxyarglabin	No ML	No ML	−104.1	Active
**1e**	Anabasinyl epoxyarglabin	No ML	No ML	−85.0	N.A.
**1f**	Anabasinyl arglabin	No ML	No ML	−82.4	N.A.
**2**	Argolide	6.46	3.69	−77.8	N.A. ^a^
**2a**	1β,10α-epoxyargolide	**4.97**	10.02	−70.5	Active
**2b**	Cytisinyl argolide	No ML	No ML	−85.2	N.A.
**2c**	Dihydroargolide	No ML	No ML	−70.7	N.A.
**3**	Grosheimin	**5.25**	7.45	−68.8	Active ^a^
**3b**	Cytisinyl grosheimin	No ML	No ML	−73.8	N.A.
**4**	3β-Hydroxyarhaline	No ML	No ML	−66.5	N.A. ^a^
**4a**	Epoxyarhaline	No ML	No ML	−80.9	N.A.
	Argracin	**4.17**	6.28	−98.7	Active ^a^
	Estafiatine	**4.12**	4.49	−65.4	Active ^a^
	Parthenolide	**4.70**	4.30	−86.4	Active ^a^

Distances indicated in bold are acceptable for covalent binding. ML, α-methylene-γ-lactone moiety. No ML, no α-methylene-γ-lactone moiety in the molecule. N.A., no activity was observed at all concentrations tested (5.125, 6.25, 25, and 50 µM). ^a^ Activities for parent compounds are as reported previously [6].

## Abbreviations

DMSO	dimethyl sulfoxide
FBS	fetal bovine serum
FPR	*N*-formyl peptide receptor
HBSS	Hanks’ balanced salt solution
ITAM	Immune-receptor tyrosine-based activation motif
TCR	T cell antigen receptor
ERK	extracellular signal-regulated kinase
MAPK	mitogen-activated protein kinases
GSH	glutathione
ELISA	enzyme-linked immunosorbent assay
